# Supplementary material for: Using machine learning to forecast peak health care service demand in real-time during the 2022–23 winter season: A pilot in England, UK
Source: PLoS One. 2025 Jan 27;20(1):e0292829. doi: 10.1371/journal.pone.0292829 (PMC11771935; doi:10.1371/journal.pone.0292829)
Supplement: S1 Table — (DOCX) [file pone.0292829.s001.docx]

# **Supporting Information**

**S1 Table 1. Forecast peak errors for each model specification.**

Key for learning methods:

ranger = random forest, kknn = k-nearest-neighbour regression, svm = support vector machine for regression, lm = linear regression, glmnet = generalised linear models with elastic net regularization, cv_glmnet = glmnet with internal optimisation of parameter lambda, xgboost = extreme gradient boosting regression, ED = emergency department; NHS = National Health Service

| **Syndromic surveillance system** | **seasonality** | **trend** | **quadratic** | **single** | **method** | **forecast peak error** |
| --- | --- | --- | --- | --- | --- | --- |
| NHS111 | Fourier | quadratic | quadratic | three_points | ranger | 0.0129 |
| NHS111 | Fourier | linear | quadratic | three_points | ranger | 0.0139 |
| NHS111 | Fourier | quadratic | quadratic | single | kknn | 0.0143 |
| NHS111 | Fourier | quadratic | linear | single | ranger | 0.0144 |
| NHS111 | Fourier | quadratic | quadratic | single | ranger | 0.0147 |
| NHS111 | months | quadratic | linear | three_points | ranger | 0.015 |
| NHS111 | months | linear | quadratic | three_points | ranger | 0.0153 |
| NHS111 | Fourier | linear | quadratic | single | kknn | 0.0157 |
| NHS111 | Fourier | linear | linear | single | ranger | 0.0158 |
| NHS111 | Fourier | linear | linear | single | kknn | 0.0158 |
| NHS111 | Fourier | quadratic | linear | three_points | ranger | 0.016 |
| NHS111 | Fourier | none | linear | single | ranger | 0.0161 |
| NHS111 | Fourier | linear | linear | three_points | ranger | 0.0166 |
| NHS111 | Fourier | quadratic | linear | single | kknn | 0.0169 |
| NHS111 | Fourier | linear | quadratic | single | ranger | 0.0169 |
| NHS111 | months | quadratic | linear | three_points | kknn | 0.0173 |
| NHS111 | months | none | quadratic | single | ranger | 0.0174 |
| ED | none | quadratic | quadratic | three_points | ranger | 0.0174 |
| NHS111 | months | quadratic | quadratic | single | kknn | 0.0177 |
| NHS111 | Fourier | none | quadratic | single | ranger | 0.0178 |
| NHS111 | months | linear | linear | single | ranger | 0.0178 |
| NHS111 | Fourier | linear | quadratic | three_points | kknn | 0.0179 |
| NHS111 | Fourier | none | quadratic | three_points | kknn | 0.018 |
| NHS111 | months | quadratic | linear | single | ranger | 0.018 |
| NHS111 | Fourier | none | quadratic | three_points | ranger | 0.018 |
| NHS111 | months | quadratic | quadratic | single | ranger | 0.0183 |
| NHS111 | months | linear | quadratic | single | ranger | 0.0188 |
| NHS111 | Fourier | linear | linear | three_points | kknn | 0.0188 |
| NHS111 | months | quadratic | quadratic | three_points | ranger | 0.019 |
| NHS111 | Fourier | none | linear | single | kknn | 0.0192 |
| NHS111 | none | quadratic | quadratic | single | ranger | 0.0192 |
| ED | Fourier | linear | quadratic | three_points | ranger | 0.0192 |
| NHS111 | months | linear | linear | three_points | ranger | 0.0193 |
| NHS111 | months | none | quadratic | three_points | ranger | 0.0194 |
| NHS111 | months | none | linear | three_points | ranger | 0.0194 |
| NHS111 | months | linear | linear | three_points | kknn | 0.0197 |
| NHS111 | Fourier | linear | quadratic | three_points | svm | 0.0197 |
| NHS111 | Fourier | quadratic | quadratic | three_points | kknn | 0.0201 |
| NHS111 | Fourier | none | linear | three_points | ranger | 0.0203 |
| NHS111 | months | none | linear | single | ranger | 0.0204 |
| NHS111 | Fourier | linear | linear | single | svm | 0.0205 |
| NHS111 | months | none | quadratic | three_points | kknn | 0.0205 |
| NHS111 | months | quadratic | quadratic | three_points | kknn | 0.0205 |
| NHS111 | Fourier | none | quadratic | single | kknn | 0.0206 |
| ED | Fourier | none | linear | single | ranger | 0.0206 |
| NHS111 | months | none | quadratic | single | kknn | 0.0207 |
| NHS111 | months | linear | quadratic | three_points | kknn | 0.0207 |
| NHS111 | Fourier | quadratic | linear | three_points | kknn | 0.0211 |
| ED | Fourier | none | linear | three_points | ranger | 0.0211 |
| NHS111 | Fourier | quadratic | quadratic | three_points | svm | 0.0213 |
| NHS111 | months | linear | quadratic | single | kknn | 0.0213 |
| NHS111 | Fourier | none | linear | three_points | kknn | 0.0213 |
| ED | Fourier | quadratic | quadratic | three_points | ranger | 0.0214 |
| NHS111 | Fourier | linear | linear | three_points | svm | 0.0214 |
| NHS111 | none | quadratic | linear | three_points | ranger | 0.0217 |
| NHS111 | months | linear | linear | single | kknn | 0.0218 |
| ED | Fourier | linear | quadratic | single | ranger | 0.0218 |
| ED | Fourier | quadratic | linear | single | ranger | 0.0219 |
| ED | Fourier | linear | linear | single | ranger | 0.0219 |
| NHS111 | Fourier | quadratic | quadratic | single | svm | 0.022 |
| NHS111 | none | linear | linear | three_points | ranger | 0.022 |
| NHS111 | none | quadratic | quadratic | three_points | ranger | 0.0224 |
| ED | Fourier | none | quadratic | three_points | ranger | 0.0224 |
| ED | none | quadratic | linear | single | ranger | 0.0229 |
| NHS111 | Fourier | quadratic | linear | three_points | svm | 0.023 |
| NHS111 | none | quadratic | linear | single | ranger | 0.0232 |
| ED | none | quadratic | quadratic | single | ranger | 0.0234 |
| NHS111 | none | linear | linear | single | ranger | 0.0236 |
| NHS111 | Fourier | linear | quadratic | single | svm | 0.0236 |
| NHS111 | Fourier | none | quadratic | three_points | svm | 0.0236 |
| NHS111 | Fourier | quadratic | linear | single | svm | 0.0236 |
| NHS111 | months | none | linear | three_points | kknn | 0.0236 |
| ED | none | quadratic | linear | three_points | ranger | 0.0238 |
| NHS111 | Fourier | none | linear | single | svm | 0.0239 |
| NHS111 | Fourier | none | quadratic | single | svm | 0.0239 |
| ED | none | linear | linear | single | ranger | 0.024 |
| NHS111 | months | quadratic | quadratic | single | svm | 0.024 |
| NHS111 | months | linear | quadratic | three_points | svm | 0.0241 |
| ED | none | linear | quadratic | single | ranger | 0.0243 |
| NHS111 | none | linear | quadratic | single | ranger | 0.0244 |
| NHS111 | months | quadratic | linear | single | kknn | 0.0246 |
| NHS111 | months | linear | linear | single | svm | 0.0246 |
| ED | months | linear | linear | single | ranger | 0.0246 |
| NHS111 | months | linear | linear | three_points | svm | 0.0246 |
| NHS111 | none | linear | quadratic | three_points | ranger | 0.0246 |
| ED | months | none | linear | three_points | ranger | 0.0246 |
| ED | months | quadratic | quadratic | three_points | kknn | 0.0247 |
| ED | Fourier | quadratic | linear | three_points | ranger | 0.0251 |
| NHS111 | Fourier | none | linear | three_points | svm | 0.0251 |
| NHS111 | months | linear | quadratic | single | svm | 0.0251 |
| ED | months | linear | linear | three_points | ranger | 0.0252 |
| ED | Fourier | quadratic | quadratic | single | ranger | 0.0253 |
| NHS111 | months | none | linear | single | kknn | 0.0253 |
| NHS111 | months | quadratic | linear | single | svm | 0.0255 |
| NHS111 | months | none | quadratic | single | svm | 0.0255 |
| NHS111 | months | quadratic | quadratic | three_points | svm | 0.0256 |
| NHS111 | none | none | linear | three_points | ranger | 0.0257 |
| NHS111 | months | quadratic | linear | three_points | svm | 0.0258 |
| ED | Fourier | none | quadratic | single | ranger | 0.026 |
| NHS111 | months | none | quadratic | three_points | svm | 0.0261 |
| ED | months | quadratic | quadratic | single | ranger | 0.0263 |
| ED | months | quadratic | linear | single | kknn | 0.0263 |
| ED | months | quadratic | linear | single | ranger | 0.0265 |
| NHS111 | months | none | linear | three_points | svm | 0.0265 |
| NHS111 | none | none | quadratic | three_points | ranger | 0.0269 |
| ED | months | quadratic | linear | three_points | kknn | 0.0269 |
| ED | Fourier | linear | linear | three_points | ranger | 0.0271 |
| NHS111 | none | none | linear | single | ranger | 0.0274 |
| NHS111 | none | quadratic | linear | three_points | kknn | 0.0277 |
| ED | months | linear | quadratic | three_points | ranger | 0.0277 |
| ED | Fourier | quadratic | quadratic | three_points | kknn | 0.0279 |
| NHS111 | Fourier | quadratic | quadratic | three_points | lm | 0.0286 |
| NHS111 | none | quadratic | quadratic | single | kknn | 0.0287 |
| NHS111 | months | linear | quadratic | single | lm | 0.0287 |
| ED | months | quadratic | quadratic | three_points | ranger | 0.029 |
| NHS111 | none | none | quadratic | single | ranger | 0.0293 |
| NHS111 | months | quadratic | quadratic | single | lm | 0.0293 |
| ED | Fourier | linear | quadratic | single | kknn | 0.0293 |
| ED | months | quadratic | linear | three_points | ranger | 0.0293 |
| ED | Fourier | none | quadratic | single | kknn | 0.0294 |
| ED | months | quadratic | linear | three_points | svm | 0.0295 |
| ED | Fourier | quadratic | quadratic | three_points | svm | 0.0296 |
| NHS111 | none | linear | linear | single | kknn | 0.0297 |
| ED | none | linear | linear | three_points | ranger | 0.0298 |
| NHS111 | months | none | linear | single | svm | 0.0298 |
| NHS111 | Fourier | quadratic | linear | three_points | lm | 0.0299 |
| NHS111 | none | quadratic | quadratic | three_points | kknn | 0.0299 |
| NHS111 | months | linear | quadratic | three_points | lm | 0.0301 |
| NHS111 | Fourier | quadratic | quadratic | single | lm | 0.0303 |
| NHS111 | months | none | linear | three_points | cv_glmnet | 0.0303 |
| NHS111 | months | linear | quadratic | three_points | cv_glmnet | 0.0303 |
| ED | none | linear | quadratic | three_points | ranger | 0.0303 |
| NHS111 | months | none | quadratic | single | cv_glmnet | 0.0303 |
| NHS111 | months | quadratic | quadratic | single | cv_glmnet | 0.0304 |
| ED | Fourier | quadratic | linear | single | kknn | 0.0304 |
| ED | months | quadratic | quadratic | single | kknn | 0.0307 |
| NHS111 | none | linear | linear | three_points | kknn | 0.0308 |
| ED | months | linear | quadratic | three_points | svm | 0.0308 |
| ED | Fourier | linear | linear | three_points | kknn | 0.0309 |
| NHS111 | months | quadratic | quadratic | three_points | lm | 0.031 |
| ED | Fourier | none | quadratic | single | svm | 0.0311 |
| NHS111 | months | quadratic | quadratic | three_points | cv_glmnet | 0.0312 |
| NHS111 | months | linear | quadratic | single | cv_glmnet | 0.0313 |
| ED | Fourier | quadratic | linear | three_points | kknn | 0.0314 |
| ED | months | none | quadratic | single | ranger | 0.0314 |
| NHS111 | Fourier | quadratic | quadratic | single | cv_glmnet | 0.0315 |
| NHS111 | Fourier | linear | quadratic | single | lm | 0.0316 |
| NHS111 | Fourier | linear | quadratic | single | cv_glmnet | 0.0317 |
| NHS111 | months | none | quadratic | single | lm | 0.0317 |
| NHS111 | Fourier | linear | quadratic | three_points | lm | 0.0318 |
| ED | Fourier | quadratic | linear | three_points | svm | 0.0318 |
| NHS111 | months | linear | linear | three_points | lm | 0.0318 |
| NHS111 | months | quadratic | linear | single | lm | 0.0319 |
| NHS111 | Fourier | none | quadratic | single | lm | 0.0321 |
| NHS111 | months | linear | linear | three_points | cv_glmnet | 0.0323 |
| NHS111 | none | linear | quadratic | three_points | kknn | 0.0324 |
| NHS111 | Fourier | none | linear | single | lm | 0.0324 |
| ED | Fourier | none | linear | single | svm | 0.0324 |
| ED | Fourier | none | quadratic | three_points | kknn | 0.0324 |
| NHS111 | Fourier | linear | linear | three_points | cv_glmnet | 0.0326 |
| ED | Fourier | quadratic | quadratic | single | kknn | 0.0328 |
| ED | Fourier | none | linear | three_points | svm | 0.0328 |
| NHS111 | none | linear | linear | three_points | svm | 0.0328 |
| NHS111 | Fourier | quadratic | linear | single | lm | 0.0328 |
| NHS111 | Fourier | quadratic | linear | three_points | cv_glmnet | 0.0329 |
| NHS111 | Fourier | linear | quadratic | three_points | cv_glmnet | 0.0329 |
| ED | months | linear | quadratic | single | svm | 0.0329 |
| ED | Fourier | linear | linear | single | kknn | 0.0331 |
| ED | Fourier | quadratic | quadratic | single | svm | 0.0331 |
| NHS111 | months | linear | linear | single | lm | 0.0331 |
| ED | months | none | linear | single | ranger | 0.0331 |
| NHS111 | none | linear | quadratic | single | kknn | 0.0332 |
| ED | Fourier | linear | quadratic | three_points | kknn | 0.0334 |
| NHS111 | months | quadratic | linear | three_points | lm | 0.0334 |
| ED | Fourier | linear | quadratic | single | svm | 0.0335 |
| NHS111 | none | none | linear | three_points | kknn | 0.0335 |
| ED | Fourier | none | linear | single | kknn | 0.0336 |
| NHS111 | months | quadratic | linear | single | cv_glmnet | 0.0336 |
| NHS111 | months | none | quadratic | three_points | lm | 0.0337 |
| NHS111 | none | none | linear | single | kknn | 0.0337 |
| ED | months | none | quadratic | single | kknn | 0.0337 |
| NHS111 | months | none | linear | single | lm | 0.0338 |
| ED | Fourier | linear | linear | single | svm | 0.0339 |
| ED | months | quadratic | quadratic | three_points | svm | 0.0339 |
| ED | months | linear | linear | three_points | svm | 0.034 |
| ED | months | linear | quadratic | single | ranger | 0.034 |
| ED | months | none | quadratic | three_points | ranger | 0.0342 |
| ED | none | none | quadratic | single | ranger | 0.0342 |
| ED | none | none | quadratic | three_points | ranger | 0.0342 |
| NHS111 | Fourier | linear | linear | single | lm | 0.0342 |
| NHS111 | none | quadratic | quadratic | three_points | svm | 0.0343 |
| NHS111 | Fourier | quadratic | linear | single | cv_glmnet | 0.0344 |
| NHS111 | months | none | quadratic | three_points | cv_glmnet | 0.0346 |
| NHS111 | none | none | quadratic | single | kknn | 0.0346 |
| NHS111 | months | linear | linear | single | cv_glmnet | 0.0347 |
| NHS111 | Fourier | none | linear | three_points | glmnet | 0.0349 |
| ED | Fourier | linear | linear | three_points | svm | 0.0349 |
| NHS111 | Fourier | none | quadratic | three_points | cv_glmnet | 0.035 |
| NHS111 | Fourier | linear | quadratic | three_points | glmnet | 0.035 |
| ED | months | linear | quadratic | three_points | kknn | 0.035 |
| NHS111 | none | linear | quadratic | single | svm | 0.0351 |
| NHS111 | months | quadratic | linear | three_points | cv_glmnet | 0.0352 |
| ED | Fourier | linear | quadratic | three_points | svm | 0.0353 |
| NHS111 | months | none | linear | three_points | lm | 0.0353 |
| NHS111 | none | linear | quadratic | three_points | svm | 0.0353 |
| NHS111 | Fourier | linear | linear | three_points | lm | 0.0353 |
| ED | Fourier | none | quadratic | three_points | svm | 0.0353 |
| ED | none | quadratic | linear | three_points | kknn | 0.0355 |
| ED | none | linear | linear | single | kknn | 0.0356 |
| NHS111 | none | quadratic | linear | single | kknn | 0.0357 |
| NHS111 | Fourier | none | linear | three_points | lm | 0.0357 |
| NHS111 | Fourier | linear | linear | single | cv_glmnet | 0.0358 |
| NHS111 | Fourier | none | quadratic | three_points | glmnet | 0.0358 |
| NHS111 | Fourier | none | linear | three_points | cv_glmnet | 0.0358 |
| NHS111 | Fourier | none | quadratic | three_points | lm | 0.036 |
| ED | Fourier | quadratic | quadratic | single | cv_glmnet | 0.036 |
| NHS111 | Fourier | quadratic | quadratic | three_points | cv_glmnet | 0.0363 |
| NHS111 | none | linear | linear | single | svm | 0.0363 |
| ED | none | linear | linear | three_points | kknn | 0.0363 |
| ED | months | quadratic | quadratic | single | svm | 0.0364 |
| NHS111 | none | none | quadratic | single | svm | 0.0365 |
| NHS111 | Fourier | none | linear | single | cv_glmnet | 0.0365 |
| ED | months | none | quadratic | single | svm | 0.0366 |
| NHS111 | months | none | linear | single | cv_glmnet | 0.0367 |
| NHS111 | Fourier | none | linear | single | glmnet | 0.0367 |
| ED | months | quadratic | linear | single | svm | 0.0367 |
| NHS111 | Fourier | none | quadratic | single | cv_glmnet | 0.0368 |
| ED | none | none | linear | three_points | ranger | 0.0368 |
| NHS111 | months | none | linear | three_points | glmnet | 0.0369 |
| NHS111 | months | none | quadratic | single | glmnet | 0.0369 |
| ED | Fourier | quadratic | quadratic | single | glmnet | 0.0371 |
| ED | months | none | linear | single | kknn | 0.0372 |
| NHS111 | none | none | quadratic | three_points | kknn | 0.0372 |
| ED | months | none | quadratic | three_points | kknn | 0.0372 |
| NHS111 | months | quadratic | quadratic | single | glmnet | 0.0374 |
| ED | none | quadratic | quadratic | three_points | kknn | 0.0375 |
| ED | months | linear | linear | three_points | kknn | 0.0377 |
| NHS111 | Fourier | quadratic | quadratic | single | glmnet | 0.0377 |
| ED | months | linear | quadratic | single | kknn | 0.0378 |
| NHS111 | none | quadratic | quadratic | three_points | lm | 0.038 |
| NHS111 | none | quadratic | quadratic | single | svm | 0.0383 |
| ED | months | linear | linear | single | svm | 0.0383 |
| NHS111 | Fourier | quadratic | linear | single | glmnet | 0.0384 |
| NHS111 | months | none | quadratic | three_points | glmnet | 0.0384 |
| NHS111 | none | quadratic | linear | three_points | svm | 0.0385 |
| ED | none | linear | quadratic | single | kknn | 0.0386 |
| ED | months | none | linear | three_points | kknn | 0.0387 |
| NHS111 | none | linear | quadratic | three_points | lm | 0.0388 |
| NHS111 | none | none | quadratic | three_points | lm | 0.0391 |
| NHS111 | months | linear | linear | three_points | glmnet | 0.0391 |
| NHS111 | months | linear | quadratic | single | glmnet | 0.0392 |
| NHS111 | Fourier | none | quadratic | single | glmnet | 0.0392 |
| NHS111 | none | none | linear | single | svm | 0.0393 |
| ED | none | none | linear | single | ranger | 0.0394 |
| NHS111 | months | linear | linear | single | glmnet | 0.0394 |
| ED | Fourier | none | linear | three_points | kknn | 0.0394 |
| NHS111 | months | quadratic | linear | single | glmnet | 0.0397 |
| ED | none | quadratic | linear | single | kknn | 0.0397 |
| NHS111 | none | quadratic | linear | single | svm | 0.0397 |
| NHS111 | Fourier | linear | linear | single | glmnet | 0.0398 |
| ED | months | linear | linear | single | kknn | 0.0398 |
| ED | Fourier | none | quadratic | single | glmnet | 0.0399 |
| ED | Fourier | none | quadratic | single | cv_glmnet | 0.0401 |
| NHS111 | Fourier | linear | linear | three_points | glmnet | 0.0402 |
| NHS111 | Fourier | linear | quadratic | single | glmnet | 0.0402 |
| NHS111 | none | linear | quadratic | single | lm | 0.0402 |
| NHS111 | Fourier | quadratic | linear | three_points | glmnet | 0.0403 |
| ED | months | none | linear | three_points | lm | 0.0403 |
| NHS111 | none | quadratic | linear | single | lm | 0.0403 |
| NHS111 | months | quadratic | linear | three_points | glmnet | 0.0404 |
| ED | Fourier | none | linear | single | lm | 0.0405 |
| ED | months | none | quadratic | three_points | svm | 0.0407 |
| ED | Fourier | none | quadratic | single | lm | 0.0407 |
| NHS111 | Fourier | quadratic | quadratic | three_points | glmnet | 0.0409 |
| ED | Fourier | linear | quadratic | single | glmnet | 0.041 |
| ED | none | quadratic | quadratic | single | kknn | 0.041 |
| ED | months | none | linear | single | svm | 0.041 |
| NHS111 | none | quadratic | quadratic | single | lm | 0.0411 |
| ED | months | none | quadratic | single | glmnet | 0.0411 |
| ED | Fourier | linear | quadratic | three_points | cv_glmnet | 0.0412 |
| NHS111 | months | linear | quadratic | three_points | glmnet | 0.0412 |
| NHS111 | none | quadratic | quadratic | single | cv_glmnet | 0.0413 |
| ED | Fourier | none | linear | three_points | glmnet | 0.0415 |
| ED | months | quadratic | quadratic | single | cv_glmnet | 0.0415 |
| NHS111 | none | quadratic | linear | three_points | lm | 0.0419 |
| ED | none | none | quadratic | three_points | kknn | 0.0419 |
| ED | Fourier | linear | quadratic | single | lm | 0.0421 |
| NHS111 | none | linear | quadratic | single | cv_glmnet | 0.0423 |
| ED | Fourier | quadratic | linear | single | svm | 0.0426 |
| ED | Fourier | linear | linear | three_points | glmnet | 0.0427 |
| NHS111 | months | none | linear | single | glmnet | 0.0428 |
| ED | none | linear | quadratic | three_points | kknn | 0.0428 |
| NHS111 | months | quadratic | quadratic | three_points | glmnet | 0.043 |
| ED | months | none | quadratic | three_points | lm | 0.0431 |
| ED | Fourier | none | quadratic | three_points | cv_glmnet | 0.0431 |
| NHS111 | none | quadratic | quadratic | three_points | cv_glmnet | 0.0432 |
| NHS111 | none | none | linear | three_points | svm | 0.0432 |
| NHS111 | none | none | quadratic | single | lm | 0.0432 |
| ED | months | linear | quadratic | three_points | lm | 0.0433 |
| ED | months | none | linear | three_points | svm | 0.0433 |
| NHS111 | none | linear | linear | three_points | lm | 0.0435 |
| NHS111 | none | linear | linear | single | glmnet | 0.0437 |
| NHS111 | none | quadratic | quadratic | single | glmnet | 0.0441 |
| ED | Fourier | linear | linear | three_points | lm | 0.0443 |
| NHS111 | none | linear | quadratic | three_points | cv_glmnet | 0.0443 |
| ED | Fourier | quadratic | quadratic | three_points | glmnet | 0.0443 |
| ED | months | quadratic | quadratic | three_points | lm | 0.0443 |
| ED | Fourier | none | linear | single | glmnet | 0.0445 |
| ED | Fourier | quadratic | linear | single | cv_glmnet | 0.0445 |
| ED | Fourier | linear | linear | single | cv_glmnet | 0.0448 |
| ED | months | linear | quadratic | single | cv_glmnet | 0.0448 |
| ED | Fourier | linear | quadratic | three_points | lm | 0.045 |
| ED | Fourier | none | linear | three_points | lm | 0.045 |
| ED | months | quadratic | quadratic | single | glmnet | 0.0452 |
| NHS111 | none | none | quadratic | single | cv_glmnet | 0.0453 |
| ED | Fourier | quadratic | linear | single | glmnet | 0.0453 |
| ED | Fourier | linear | linear | single | glmnet | 0.0454 |
| ED | months | linear | linear | single | glmnet | 0.0454 |
| ED | Fourier | quadratic | quadratic | single | lm | 0.0454 |
| NHS111 | none | none | quadratic | three_points | cv_glmnet | 0.0454 |
| ED | months | none | quadratic | single | lm | 0.0454 |
| ED | Fourier | quadratic | linear | three_points | lm | 0.0454 |
| ED | none | none | quadratic | single | kknn | 0.0454 |
| NHS111 | none | none | linear | three_points | lm | 0.0458 |
| ED | Fourier | none | quadratic | three_points | lm | 0.0458 |
| ED | none | none | quadratic | three_points | svm | 0.0459 |
| ED | none | linear | linear | single | svm | 0.0459 |
| ED | months | quadratic | quadratic | three_points | cv_glmnet | 0.0459 |
| NHS111 | none | none | linear | single | lm | 0.0459 |
| NHS111 | none | quadratic | linear | single | glmnet | 0.046 |
| NHS111 | none | none | quadratic | three_points | svm | 0.046 |
| NHS111 | none | quadratic | linear | three_points | cv_glmnet | 0.0461 |
| ED | months | none | linear | single | cv_glmnet | 0.0463 |
| ED | none | linear | linear | three_points | svm | 0.0463 |
| ED | Fourier | quadratic | quadratic | three_points | lm | 0.0464 |
| ED | months | quadratic | linear | three_points | lm | 0.0464 |
| NHS111 | none | linear | quadratic | single | glmnet | 0.0466 |
| NHS111 | none | linear | linear | single | lm | 0.0466 |
| ED | months | none | quadratic | single | cv_glmnet | 0.0466 |
| ED | none | none | quadratic | single | lm | 0.0467 |
| ED | Fourier | none | linear | single | cv_glmnet | 0.0467 |
| ED | none | linear | quadratic | three_points | glmnet | 0.0468 |
| ED | none | quadratic | quadratic | single | cv_glmnet | 0.0468 |
| NHS111 | none | none | quadratic | three_points | glmnet | 0.047 |
| ED | Fourier | quadratic | linear | three_points | cv_glmnet | 0.0471 |
| NHS111 | none | none | linear | three_points | glmnet | 0.0471 |
| ED | months | linear | quadratic | single | glmnet | 0.0473 |
| ED | months | quadratic | linear | single | lm | 0.0473 |
| ED | none | none | linear | single | glmnet | 0.0473 |
| ED | months | none | quadratic | three_points | cv_glmnet | 0.0473 |
| ED | Fourier | linear | linear | three_points | cv_glmnet | 0.0474 |
| NHS111 | none | quadratic | linear | three_points | glmnet | 0.0474 |
| ED | Fourier | linear | quadratic | single | cv_glmnet | 0.0474 |
| ED | Fourier | quadratic | linear | three_points | glmnet | 0.0476 |
| ED | none | none | quadratic | three_points | lm | 0.0477 |
| ED | months | linear | linear | three_points | glmnet | 0.0479 |
| NHS111 | none | linear | linear | three_points | glmnet | 0.0479 |
| NHS111 | none | quadratic | linear | single | cv_glmnet | 0.0482 |
| ED | months | linear | linear | three_points | lm | 0.0483 |
| ED | none | none | quadratic | single | svm | 0.0484 |
| ED | none | quadratic | linear | single | svm | 0.0484 |
| ED | months | quadratic | linear | three_points | cv_glmnet | 0.0486 |
| ED | months | quadratic | linear | single | cv_glmnet | 0.0487 |
| ED | months | quadratic | linear | three_points | glmnet | 0.0487 |
| ED | months | quadratic | quadratic | single | lm | 0.0489 |
| NHS111 | none | linear | linear | three_points | cv_glmnet | 0.049 |
| ED | months | none | linear | single | glmnet | 0.0491 |
| ED | none | quadratic | quadratic | three_points | lm | 0.0492 |
| NHS111 | none | linear | linear | single | cv_glmnet | 0.0493 |
| ED | months | quadratic | quadratic | three_points | glmnet | 0.0494 |
| ED | months | none | linear | three_points | glmnet | 0.0494 |
| ED | none | quadratic | quadratic | single | glmnet | 0.0496 |
| ED | months | linear | quadratic | single | lm | 0.0497 |
| ED | none | linear | quadratic | three_points | svm | 0.0497 |
| ED | Fourier | linear | quadratic | three_points | glmnet | 0.0498 |
| NHS111 | none | none | linear | single | glmnet | 0.0499 |
| ED | none | quadratic | linear | three_points | svm | 0.0499 |
| ED | Fourier | quadratic | linear | single | lm | 0.0499 |
| ED | months | linear | linear | single | cv_glmnet | 0.0502 |
| ED | none | none | linear | three_points | glmnet | 0.0503 |
| ED | none | linear | linear | three_points | lm | 0.0505 |
| NHS111 | none | none | quadratic | single | glmnet | 0.0505 |
| ED | none | linear | linear | single | cv_glmnet | 0.0505 |
| NHS111 | none | linear | quadratic | three_points | glmnet | 0.0506 |
| ED | months | quadratic | linear | single | glmnet | 0.0506 |
| ED | none | linear | quadratic | single | cv_glmnet | 0.0506 |
| ED | none | linear | quadratic | single | svm | 0.0507 |
| ED | months | none | linear | three_points | cv_glmnet | 0.0509 |
| NHS111 | none | none | linear | three_points | cv_glmnet | 0.051 |
| ED | none | none | linear | three_points | kknn | 0.0511 |
| ED | none | quadratic | quadratic | three_points | svm | 0.0511 |
| ED | Fourier | linear | linear | single | lm | 0.0511 |
| ED | none | quadratic | quadratic | single | lm | 0.0513 |
| ED | none | none | linear | single | svm | 0.0513 |
| ED | months | linear | linear | single | lm | 0.0514 |
| ED | none | quadratic | linear | single | glmnet | 0.0514 |
| NHS111 | none | quadratic | quadratic | three_points | glmnet | 0.0514 |
| ED | months | linear | quadratic | three_points | cv_glmnet | 0.0517 |
| ED | months | linear | quadratic | three_points | glmnet | 0.0517 |
| ED | none | none | linear | three_points | lm | 0.0517 |
| ED | none | linear | linear | single | glmnet | 0.052 |
| ED | Fourier | none | linear | three_points | cv_glmnet | 0.052 |
| ED | none | quadratic | linear | single | lm | 0.052 |
| ED | none | linear | quadratic | single | lm | 0.052 |
| ED | months | linear | linear | three_points | cv_glmnet | 0.052 |
| ED | Fourier | quadratic | quadratic | three_points | cv_glmnet | 0.0526 |
| ED | none | quadratic | quadratic | three_points | cv_glmnet | 0.0529 |
| ED | months | none | quadratic | three_points | glmnet | 0.0532 |
| ED | none | none | linear | three_points | svm | 0.0533 |
| ED | none | quadratic | quadratic | single | svm | 0.0535 |
| ED | Fourier | none | quadratic | three_points | glmnet | 0.0537 |
| ED | none | none | quadratic | three_points | cv_glmnet | 0.0537 |
| ED | none | none | linear | single | cv_glmnet | 0.0539 |
| NHS111 | none | none | linear | single | cv_glmnet | 0.054 |
| ED | none | linear | quadratic | single | glmnet | 0.054 |
| ED | none | none | linear | three_points | cv_glmnet | 0.054 |
| ED | none | linear | quadratic | three_points | cv_glmnet | 0.0542 |
| ED | none | quadratic | linear | single | cv_glmnet | 0.0544 |
| ED | none | quadratic | linear | three_points | lm | 0.0548 |
| ED | none | none | quadratic | single | cv_glmnet | 0.0554 |
| ED | none | quadratic | linear | three_points | cv_glmnet | 0.0554 |
| ED | months | none | linear | single | lm | 0.0559 |
| ED | none | quadratic | quadratic | three_points | glmnet | 0.0573 |
| ED | none | linear | linear | three_points | glmnet | 0.0577 |
| ED | none | linear | quadratic | three_points | lm | 0.0579 |
| ED | none | none | quadratic | single | glmnet | 0.058 |
| ED | none | none | linear | single | kknn | 0.058 |
| ED | none | quadratic | linear | three_points | glmnet | 0.0581 |
| ED | none | none | linear | single | lm | 0.0582 |
| ED | none | none | quadratic | three_points | glmnet | 0.0604 |
| ED | none | linear | linear | single | lm | 0.0616 |
| ED | none | linear | linear | three_points | cv_glmnet | 0.0628 |
| NHS111 | Fourier | quadratic | quadratic | three_points | xgboost | 0.11 |
| NHS111 | months | linear | linear | single | xgboost | 0.1181 |
| NHS111 | months | linear | quadratic | three_points | xgboost | 0.1191 |
| NHS111 | Fourier | linear | linear | single | xgboost | 0.1198 |
| NHS111 | months | quadratic | quadratic | single | xgboost | 0.1199 |
| NHS111 | months | linear | linear | three_points | xgboost | 0.1227 |
| NHS111 | months | none | linear | single | xgboost | 0.1234 |
| NHS111 | Fourier | quadratic | linear | three_points | xgboost | 0.1241 |
| NHS111 | Fourier | quadratic | quadratic | single | xgboost | 0.1242 |
| NHS111 | Fourier | linear | quadratic | single | xgboost | 0.1256 |
| NHS111 | Fourier | linear | quadratic | three_points | xgboost | 0.1264 |
| NHS111 | Fourier | none | linear | single | xgboost | 0.1268 |
| NHS111 | months | quadratic | linear | single | xgboost | 0.1282 |
| NHS111 | Fourier | quadratic | linear | single | xgboost | 0.1282 |
| NHS111 | months | quadratic | quadratic | three_points | xgboost | 0.1284 |
| NHS111 | months | none | linear | three_points | xgboost | 0.1285 |
| NHS111 | months | quadratic | linear | three_points | xgboost | 0.1286 |
| NHS111 | months | none | quadratic | three_points | xgboost | 0.1292 |
| NHS111 | months | linear | quadratic | single | xgboost | 0.1296 |
| NHS111 | Fourier | none | quadratic | single | xgboost | 0.1301 |
| NHS111 | Fourier | linear | linear | three_points | xgboost | 0.1302 |
| NHS111 | Fourier | none | linear | three_points | xgboost | 0.1325 |
| NHS111 | none | quadratic | quadratic | three_points | xgboost | 0.1336 |
| NHS111 | Fourier | none | quadratic | three_points | xgboost | 0.1351 |
| NHS111 | none | linear | linear | single | xgboost | 0.1364 |
| NHS111 | months | none | quadratic | single | xgboost | 0.1368 |
| NHS111 | none | linear | linear | three_points | xgboost | 0.1375 |
| NHS111 | none | linear | quadratic | three_points | xgboost | 0.1376 |
| NHS111 | none | quadratic | quadratic | single | xgboost | 0.1382 |
| NHS111 | none | quadratic | linear | single | xgboost | 0.1398 |
| ED | months | none | linear | single | xgboost | 0.14 |
| ED | Fourier | linear | linear | single | xgboost | 0.1402 |
| NHS111 | none | quadratic | linear | three_points | xgboost | 0.1408 |
| ED | months | linear | linear | single | xgboost | 0.1408 |
| NHS111 | none | linear | quadratic | single | xgboost | 0.1421 |
| NHS111 | none | none | quadratic | three_points | xgboost | 0.1422 |
| NHS111 | none | none | quadratic | single | xgboost | 0.1425 |
| NHS111 | none | none | linear | three_points | xgboost | 0.1434 |
| NHS111 | none | none | linear | single | xgboost | 0.144 |
| ED | none | linear | linear | single | xgboost | 0.1455 |
| ED | Fourier | none | linear | single | xgboost | 0.1498 |
| ED | none | none | linear | single | xgboost | 0.1733 |
