# Supplementary material for: Using machine learning to forecast peak health care service demand in real-time during the 2022–23 winter season: A pilot in England, UK
Source: PLoS One. 2025 Jan 27;20(1):e0292829. doi: 10.1371/journal.pone.0292829 (PMC11771935; doi:10.1371/journal.pone.0292829)
Supplement: Attachment — Submitted filename: PONE-D-23-30068-response to reviewers.docx [file pone.0292829.s002.docx]

Dear Editor

Thank you to the reviewers for their feedback – the reviews were very constructive and we believe they have greatly improved our manuscript. We have considered each of the reviewers reports and below we provide a point-by-point response. We have amended our manuscript accordingly.

We have also made the following formatting amendments requested by the journal:

- We have amended our manuscript throughout to meet PLOS ONE’s stye requirements.
- The funding statement has been removed from the manuscript (see below).
- The training data used for this study has been uploaded to a public repository (*/roger.morbey@bvbrc/Syndromic RSV indicators for ML forecasting*) and is included in the data availability statement (see below).
- The figures have been removed from the main body of the manuscript and uploaded as separate files.
- The tables have been moved to the section of the manuscript where they are first referenced.
- The caption for the supplementary table has now been added to the end of the manuscript.

The funding statement has been removed from the manuscript file but needs to be included in the publication:

**Funding:** RAM, DT and AJE are affiliated with the National Health Institute for Health Care Research (NIHR) Health Protection Research Unit (HPRU) in Emergency Preparedness and Response at King's College London. AJE is affiliated with the NIHR HPRU in Gastrointestinal Infections at University of Liverpool. DT is supported by the NIHR Applied Research Collaboration (ARC) West Midlands. The views expressed are those of the author(s) and not necessarily those of the NIHR, the UK Health Security Agency or the UK Department of Health and Social Care.

**Data availability statement:**

Data used to train our models is publically available at: */roger.morbey@bvbrc/Syndromic RSV indicators for ML forecasting*

We look forward to hearing from you.

Best wishes

Roger Morbey

**Response to reviewers**

**Reviewer #1**

The authors piloted a new forecasting pipeline during the Winter of 2022-23, using existing surveillance indicators which are sensitive to increases in respiratory syncytial virus (RSV). Indicators including telehealth cough calls and ED bronchiolitis attendances, both in children under 5 years. They utilised machine learning techniques to train and select models that would best forecast the timing and intensity of peaks up to 28 days ahead. Forecast uncertainty was modelled usings a novel gamlss approach which enabled prediction intervals to vary according to the level of the forecast activity.

They calculated forecast reliability to describe uncertainty around forecasts and piloted forecasts during the 2022-23 winter season and compared forecasts to actual activity.

They have demonstrated the utility of the approach, adding forecasts to existing surveillance systems.

Response: we thank the reviewer their comments.

**Reviewer #2**

Authors proposed "Using machine learning to forecast peak health care service demand in real-time during the 2022-23 winter season: a pilot in England, UK". The structure of the article is well structured but authors should consider the following comments.

1. Proofread the entire manuscript.

2. Draw a graphical abstract of your proposed approach

3. compare your approach with previous existing approaches

Response: We have proofread the manuscript and included the formatting changes requested by the editor to ensure compliance with the journal style. Figure 1 provides an overview of the processes involved in our forecasting; we are happy for this to be used as a graphical abstract if required by the journal format.

We have added a new section to the discussion (4.5 Comparison with existing approaches) which now provides a comparison with existing forecasting approaches. We have supported this new discussion with 5 new references.

**Reviewer #3**

a) Language

Minor language errors such as "Derived variables were created that were used to create the forecast models."

Response: We have made corrections to improve the grammar and readability of the paper.

Some abbreviations used before or without definition: gamlss.

Response: We have spelt out gamlss and included a reference. We have also checked throughout the manuscript for proper formatting of other abbreviations.

b) Methods

The methods should be more thoroughly explained and the sections should better structured.

Response: We have expanded the methods section as explained below, including making the structure of the model specifications clearer through the combination of different machine learning methods and model options for which derived variables to include.

The current state of the paper does not permit reproducing the results or even applying the proposed solution to other data, because of the lack of details in what the methods are concerned. Some examples:

- why were only two syndromic indicators used. Did the authors try others as well? What is the justification for choosing those two?

Response: We will make the data used in our analysis publicly available. We can also make the scripts we used for the analysis available to enable others to reproduce our results or apply the same methods to other data.

In this pilot forecast we focussed on forecasting RSV using two of the most established indicators available in our routine surveillance. However, we have developed our pipelines and methodology so it will be easy to extend this work to other indicators and it is our intention to consider influenza-like-illness and other respiratory indicators in future work.

- the authors state that they used a smoothing method to account for the effects of holidays and weekend and simply reference an article without giving any other detail. The paper should be self-contained, meaning they need to at least mention what kind of smoothing method they use, and, possible particularities with respect to their data, if any.

Response: We have added more detail about our approach to account for day of the week effects.

- the authors state: "For each of these seven different methods we included the following options:" and proceed to enumerate 4 such options. These options are in fact data preprocessing methods - as far as I can tell, should be listed as such and should be better explained. This part requires serious review.

Response: The reviewer is correct that these options are introduced part of the preprocessing. However, they do create distinct models, for instance whether or not seasonality is included in a model. We have expanded this section to make clearer what we mean by different methods and different models.

What does "With or without a quadratic term for current activity" mean? To what current activity is this referring to and how exactly (formally) is this used?

Response: We have expanded our explanation of the derived variables and the options we used. We used an optional additional variable that was the square of daily activity so that we could compare models that had a linear or a quadratic relationship with daily activity.

- the authors first describe the data, then the methods, then they come back to describing the data (see, for example, paragraph starting with "We used anonymised health service data that is routinely used by UKHSA for public health surveillance of respiratory illnesses, including RSV."). The section should be structured for better readability.

Response: Thank you for this suggestion we have moved this sentence to section 2.1 on data selection.

- "The forecast peak error includes weighting to emphasise the importance of accurately forecasting peaks when activity is high." What values were used for the weights? This is an important detail that should also be motivated, let alone mentioned: is it 0.5 for both? Why?

Response: Apologies for not including this important measure, which we had published in a previous paper. We have now reproduced the full details on how this is calculated along with the original citation.

- "a gamlss model was used to estimate standard deviation variation against the level of current activity." What kind of distribution.

Response: We have included the citation for the original work on gamlss.

[end]
